# Effects of Curing Distance and Time on the Microhardness of Posterior Composites: An Experimental Study

**DOI:** 10.7759/cureus.96319

**Published:** 2025-11-07

**Authors:** Rajinder Bansal, Om Prakash Pandey, Vishakha Grover, Poonam Bogra, Saru Gupta, Saurabh Gupta, Seema Gupta

**Affiliations:** 1 Department of Conservative Dentistry and Endodontics, Guru Nanak Dev Dental College and Research Institute, Sunam, IND; 2 Department of Physics and Material Science, Thapar Institute of Engineering and Technology, Patiala, IND; 3 Department of Periodontology, Dr. Harvansh Singh Judge Institute of Dental Sciences and Hospital, Chandigarh, IND; 4 Department of Conservative Dentistry and Endodontics, J.N. Kapoor, D.A.V Centenary Dental College, Yamunanagar, IND; 5 Department of Pediatric and Preventive Dentistry, Maharishi Markandeshwar College of Dental Sciences and Research, Ambala, IND; 6 Department of Conservative Dentistry and Endodontics, All India Institute of Medical Sciences, Jammu, IND; 7 Department of Orthodontics, Kothiwal Dental College and Research Centre, Moradabad, IND

**Keywords:** composite resins, curing, distance, microhardness, polymerization, time

## Abstract

Introduction

Composite resins are commonly employed in restorative dentistry because of their aesthetic and functional properties, with microhardness serving as a key measure of their durability and clinical success. The degree of polymerization of the composites may be influenced by variables such as the distance between the curing tip and resin surface, as well as the duration of light exposure. Therefore, this in vitro study aimed to evaluate the combined effects of varying curing tip distances and exposure times on the microhardness of a posterior composite resin, providing insights into optimizing clinical protocols for enhanced restoration longevity.

Materials and methods

This in vitro study was conducted in the Department of Conservative Dentistry and Endodontics and used SureFil™ (Dentsply Sirona, Charlotte, NC, USA), a packable posterior composite with an 82% filler loading. Sixty cylindrical samples (6 mm diameter and 5 mm thickness) were prepared in a two-part split mold. Four groups (A, B, C, and D) were defined by curing tip distances of 1, 3, 5, and 8 mm, respectively, using metal spacer plates. Each group was subdivided into three subgroups based on exposure times of 40 s, 60 s, and 80 s, with five samples per subgroup. The samples were cured using a halogen curing unit (DENTSPLY®Caulk QHL75™, 450 mW/cm²). After 24 hours at 100% humidity, the samples were embedded in acrylic, polished, and tested for microhardness using a Mitutoyo HM-200 tester (Mitutoyo Corporation, Kawasaki, Japan) at depths ranging from 0.5 to 5.0 mm. The Vickers hardness number (VHN) was calculated, and the data were analyzed using two-way analysis of variance and Bonferroni post-hoc tests.

Results

Microhardness decreased significantly with increasing curing distance (p < 0.001) and was highest at 1 mm with 80 s of curing, whereas the lowest values were at 8 mm for 40 s. Longer exposure times (80 s) improved the microhardness across all distances, particularly at 5 mm. The curing distance explained 96% of the variance (p < 0.001), with a non-significant interaction effect (p = 0.065).

Conclusion

Minimizing the curing tip distance (1-3 mm) and extending the exposure time (≥60 s) significantly enhanced the microhardness of posterior composite resins, optimized polymerization, and improved clinical durability.

## Introduction

Composite resins have become the cornerstone of modern restorative dentistry because of their aesthetic properties, versatility, and ability to bond effectively to tooth structures. These materials, composed of a resin matrix and inorganic fillers, are polymerized via light-curing processes to achieve the desired mechanical and physical properties [1]. Among these properties, microhardness is a critical parameter because it reflects the resistance of a material to surface deformation and wear, directly influencing its clinical durability and performance [2]. The microhardness of composite resins is significantly influenced by the polymerization process, which is governed by many factors, including the composition of resin-based composites, mode of curing, duration of light curing, thickness of increments, types of light-curing units employed, post-irradiation duration, dimensions and positioning of the cavity, proximity of the light-curing tip to the surface, substrate material, filler type, and temperature conditions [3,4]. Understanding the interplay between these variables is essential for optimizing clinical outcomes and ensuring the longevity of restorations.

The light-curing process involves the use of a light-curing unit, typically employing light-emitting diodes or halogen lights, to initiate polymerization. The effectiveness of this process depends on the delivery of an adequate light intensity to the composite resin surface [5]. The curing tip distance, defined as the distance between the light-curing tip and the resin surface, plays a pivotal role in determining the intensity of light reaching the material. As the distance increases, the light intensity decreases, owing to the inverse square law, potentially leading to incomplete polymerization and compromised material properties, including reduced microhardness [6]. Previous studies have demonstrated that greater curing tip distances can result in lower degrees of conversion, which is directly correlated with reduced mechanical strength and surface hardness [6,7].

The exposure time, that is, the duration for which the composite resin is exposed to the light-curing unit, is another critical factor. Adequate exposure ensures sufficient energy delivery to initiate and sustain the polymerization reaction, thereby promoting cross-linking within the resin matrix [4]. An insufficient exposure time may lead to incomplete curing, resulting in a softer material prone to wear, degradation, and secondary caries [4]. Therefore, an increased curing time of 60 s has been recommended in a previous study for increased curing distance in Class II composite restorations covering the gingival floor of deep cavities of up to 8 mm [8]. Conversely, excessive exposure may not yield proportional benefits and could potentially cause thermal damage to the surrounding tooth structure or pulp [9].

In vitro studies have provided a controlled environment to systematically investigate the effects of curing parameters on composite resin properties. Such studies are crucial for isolating variables such as curing tip distance and exposure time, which are often confounded by clinical factors such as operator technique, saliva contamination, and patient movement [4,5,7]. By evaluating microhardness, a reliable indicator of polymerization quality, these studies offer insights into how curing conditions can be optimized to enhance the clinical performance of composite resins [7]. Recent advancements in composite formulations and light-curing unit technology have necessitated updated investigations to establish evidence-based guidelines for practitioners.

This study aimed to evaluate the combined effects of curing tip distance and exposure time on the microhardness of posterior composite resins in an in vitro setting. The findings offer evidence-based recommendations to refine clinical practice, aiming to improve the durability and success of posterior composite restorations.

## Materials and methods

Study design

This in vitro study was conducted in the Department of Physics and Material Science, Thapar Institute of Engineering and Technology for a span of six months. Ethical approval was not required for this study because it was conducted in vitro and did not involve human or animal subjects.

The study design involved a factorial approach, systematically varying curing tip distances (1, 3, 5, and 8 mm) and exposure times (40, 60, and 80 s) to assess their impact on the Vickers hardness number (VHN) of a standard posterior composite resin. The composite resin selected was SureFil™ (Dentsply Sirona, Charlotte, NC, USA), a packable low-viscosity ‘flowable’ bulk fill posterior composite resin with a filler loading of approximately 82% and an average filler particle size of 0.8 microns, composed of barium fluoro-alumino borosilicate glasses and fumed silica. This material is commonly used for Class II restorations owing to its high strength and wear resistance [10]. SureFil™ was placed in two 2.5 mm increments, each light-cured separately through the spacer plate with the halogen curing tip centered over the samples. The samples were cured using a halogen curing unit (DENTSPLY®Caulk QHL75™, 450 mW/cm²). Rueggeberg et al. recommended a light source intensity of at least 400 mW/cm2 [11].

Sample size estimation

The sample size was determined using G*Power software (version 3.1.9.2, Heinrich Heine University, Düsseldorf, Germany) with an effect size of 0.44, derived from a prior study analyzing the relationship between curing time and microhardness in posterior composite restorations [4]. To achieve 80% statistical power and a 5% alpha error threshold, 60 samples were included in the study. These samples were equally distributed into four groups (n = 15 per group), stratified by the distance between the curing tip and composite surface.

Inclusion criteria

To be included, samples had to be cylindrical in shape (6mm x 5mm), made from SureFil™ resin, and must have been fully cured using the correct light for a specific time and distance. They also had to be mounted in acrylic and polished to a very smooth, shiny surface before testing. Samples were excluded if they exhibited voids, cracks, or physical defects from preparation or any contamination. Additionally, any sample cured when the light unit’s irradiance fell outside the verified range of 400-500 mW/cm² was also excluded to ensure consistent polymerization conditions.

Methodology

**Figure 1:**
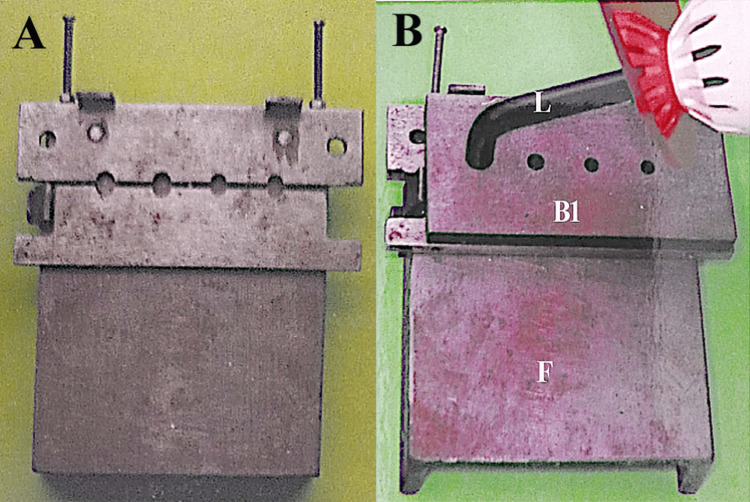
(A) Custom metal device with corresponding parts, (B) metal device with curing light Image credit: Dr Rajinder Bansal.

A custom metal device was fabricated for curing the composite from different distances (Figure 1).

**Figure 2:**
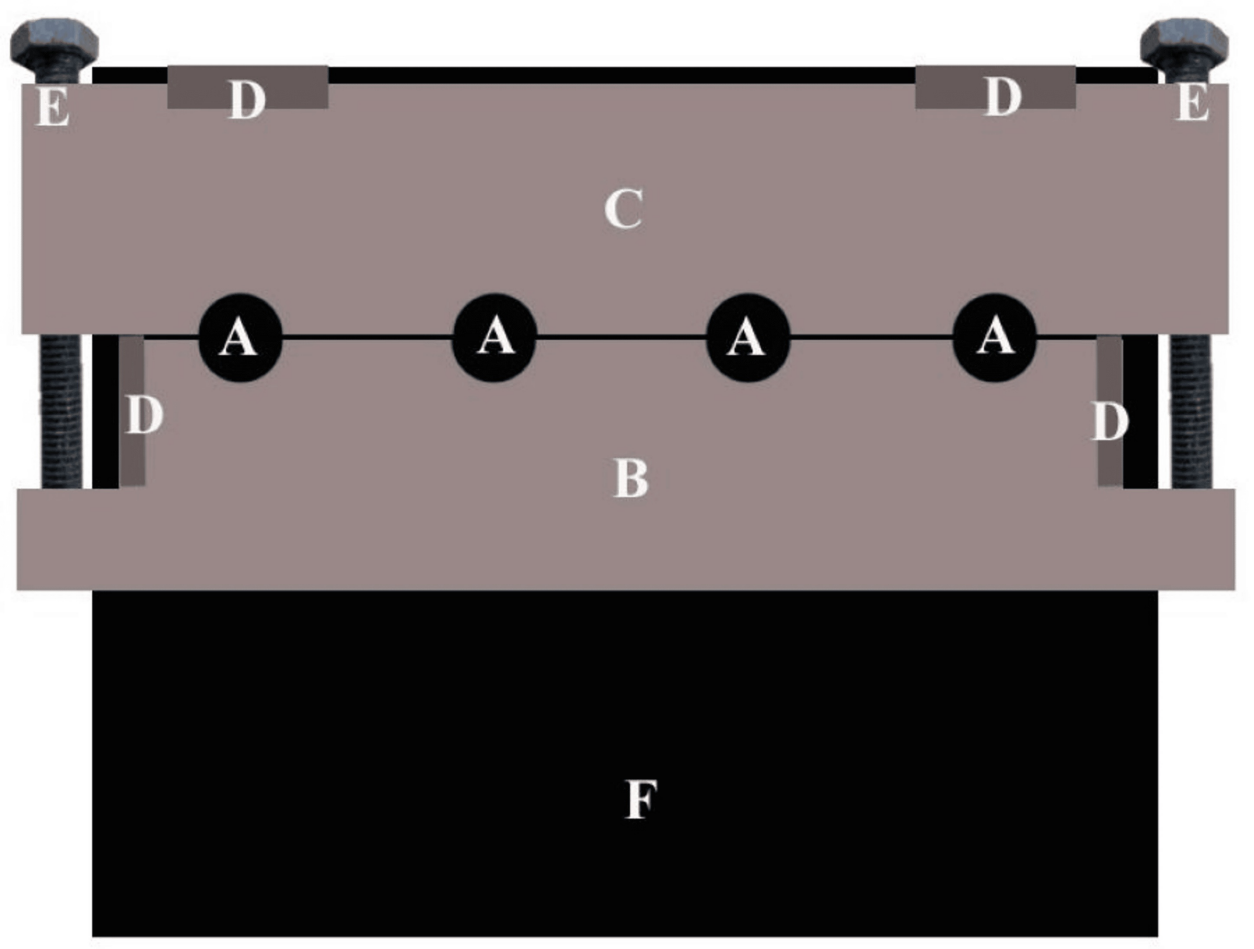
Schematic representation of the custom metal fabrication device used for preparing composite cylinders (6 mm diameter × 5 mm thickness). Components include: (A) Composite cylinder samples, (B) Sliding part, (C) Fixed part, (D) Clamps, (E) Adjustable bolts, (F) Metal base plate (10 × 10 cm). The device features four wells (presented as "A" of dimension 6 mm diameter × 5 mm thickness) at the junction of B and C to house composite material. Original image created by Dr. Rajinder Bansal

A custom metal base plate (F, 10 × 10 cm) was fabricated (Figure 1) to hold two primary components: a fixed part (C) and a sliding part (B), secured with clamps (D) and adjustable bolts (E). At the junction of B and C, four wells (presented as "A" of dimension 6 mm diameter × 5 mm thickness) were machined to contain composite material. The composite material was packed into each well, and the assembly was tightened uniformly using bolts (E) to minimize voids. For a 1 mm curing distance, a light-curing tip was placed 1 mm atop the composite surface and activated for 40 s (standard curing time). To simulate curing distances of 3 mm, 5 mm, and 8 mm, precision metal spacer plates of corresponding thicknesses, each with wells matching the dimensions of those in the base plate, were positioned between the light-curing tip and composite surface, ensuring consistent alignment. The light-curing unit’s irradiance was verified with a radiometer before each use. After curing, composite cylinders (6 mm diameter × 5 mm thickness) were extracted and mounted on acrylic blocks for stabilization (Figure 2).

**Figure 3:**
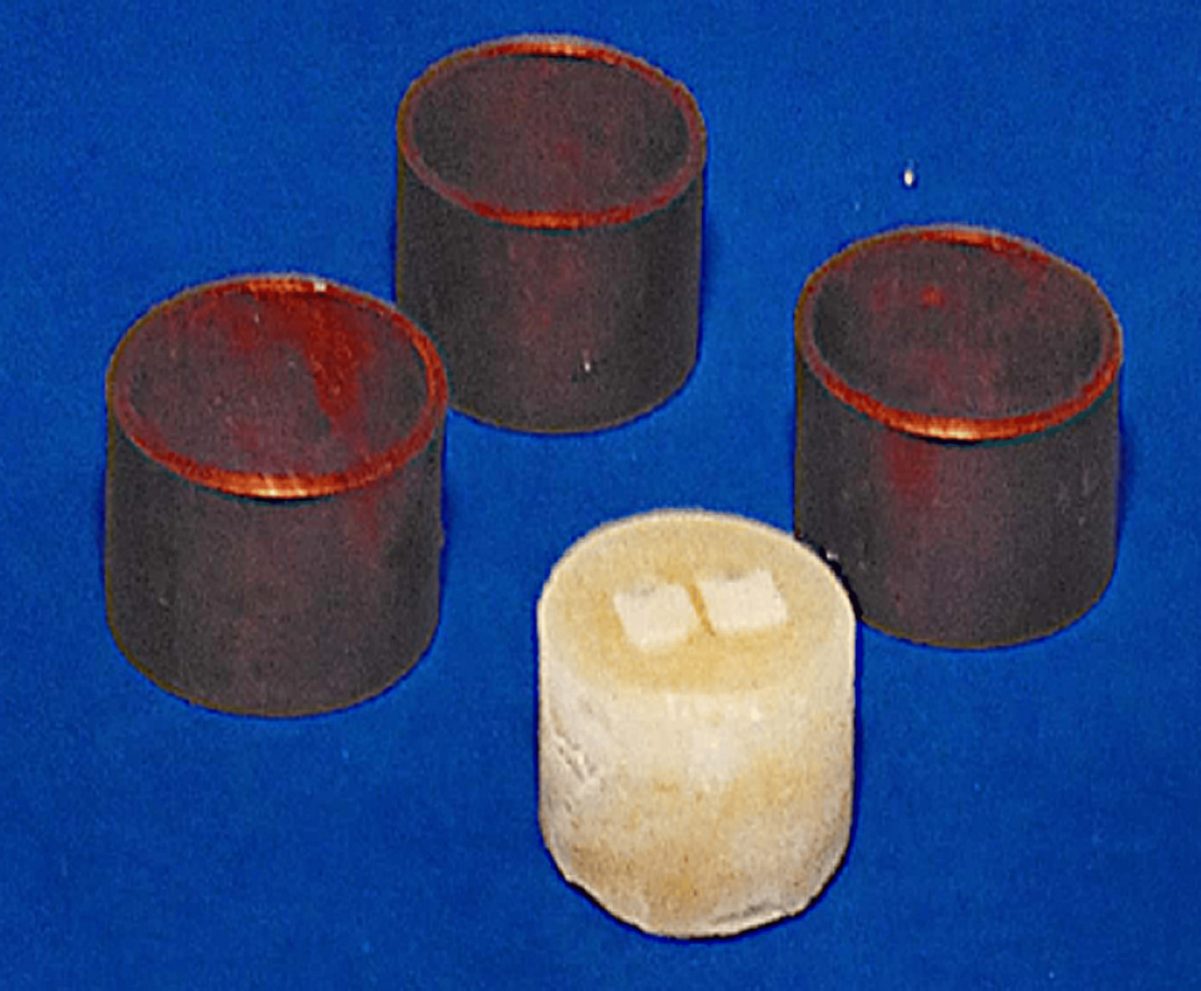
Cylindrical molds to prepare the acrylic base for testing the Vickers hardness number of the composite Image credit: Dr Rajinder Bansal

Sixty cylindrical samples (6 mm diameter × 5 mm thickness) were prepared to typically simulate posterior composite restorations in height and width (Figure 3), ensuring clinical relevance for evaluating the microhardness of composite resins [12]. The study samples were divided into four groups (A, B, C, and D) based on the curing tip distances of 1, 3, 5, and 8 mm. Each group was further subdivided into 3 subgroups based on exposure times of 40, 60, and 80 s, with 5 samples per subgroup, resulting in 15 samples per group.

**Figure 4:**
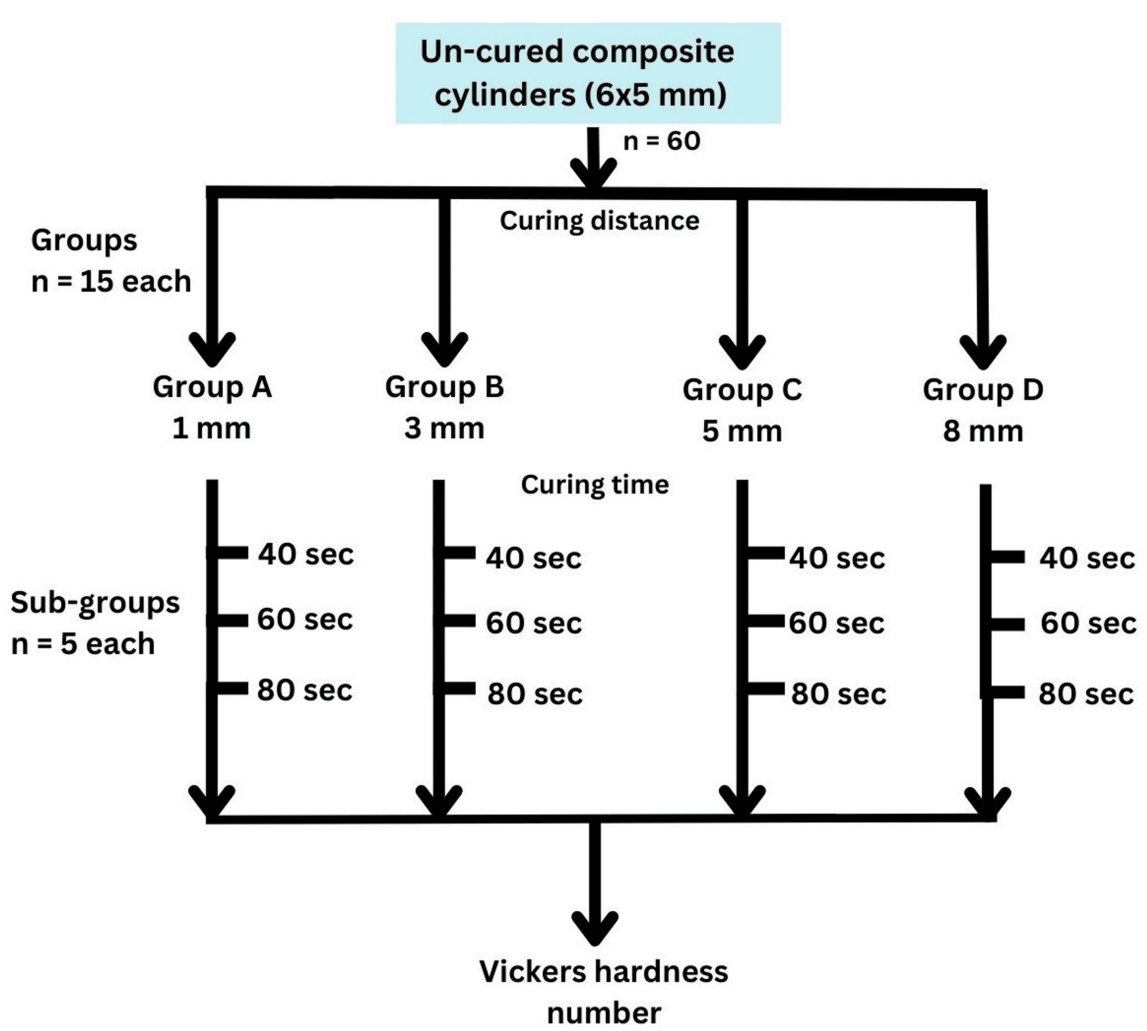
Study flowchart

The flowchart of the study is shown in Figure 4.

To prepare the samples, the mold was disassembled, and a Mylar strip (Dentalink, Mumbai, India) was placed between the two halves of each cylindrical cavity to facilitate separation. The SureFil™ composite (lot 981116, shade A) was incrementally packed into half-cylinders using a Teflon-coated instrument (Ash Ceramicolor, Dentsply Sirona, Charlotte, NC, USA). After filling, a Mylar strip was placed on the top surface and pressed with the thumb to ensure a smooth and uniform surface, and excess material (flash) was removed using a scalpel. An appropriate metal spacer plate was placed on the mold to set the curing tip distance, and the composite was cured using a light-curing unit, with the curing tip positioned directly on the spacer plate. For each subgroup, five samples were cured at specified exposure times (40, 60, or 80 s). After curing, the mold was disassembled, and semi-circular resin halves were marked to distinguish between the top and bottom surfaces.

All samples were stored at 100% humidity and 370° C for 24 hours to simulate oral conditions and allow for post-cure polymerization. Each semi-circular sample was then embedded in a hollow copper tube using self-cure acrylic resin (Ashvin, Delhi, India). Vaseline (Pond India Limited, Mumbai, India) was applied to the inner surface of the copper tubes to facilitate the removal of acrylic blocks. The flat surface of each embedded sample was oriented downward, and the curved surface was encapsulated in acrylic. After the acrylic set, the samples were removed from the tubes and subjected to a standardized polishing protocol. The initial grinding was performed using an automated polishing unit (Buehler, Lake Bluff, IL, USA), followed by hand polishing with progressively finer emery papers (600, 800, and 1200 grit). Final polishing was achieved with an aqueous solution of submicron alumina powder on a second automated polishing unit (Buehler, Lake Bluff, IL, USA) to ensure a smooth reflective surface suitable for microhardness testing.

Outcome measurement

The microhardness was measured using a microhardness tester (Mitutoyo HM-200, Mitutoyo Corp., Kawasaki, Japan). A 25-gram load was applied for 10 s to create indentations at depths of 0.5 mm, 1.0 mm, 1.5 mm, 2.0 mm, 2.5 mm, 3.0 mm, 3.5 mm, 4.0 mm, 4.5 mm, and 5.0 mm from the top surface of each sample. Three indentations were made at each depth to yield 30 measurements per sample. VHN was calculated using the formula: VHN = 1.854 × P/D², where P is the applied load in kilogram-force (kgf) and D is the mean diagonal length of the indentation (mm). The mean VHN at each depth was computed from three measurements.

To ensure reliability, the microhardness tester was calibrated before testing using a standard reference block provided by Mitutoyo. Calibration was verified by measuring the VHN of the reference block at a 25-gram load, ensuring that measurements fell within the manufacturer’s specified range. Inter-examiner reliability was assessed by two trained operators independently measuring VHN on a subset of 10 randomly selected samples. Intraclass correlation coefficients (ICC) were calculated, yielding an ICC of 0.92, indicating high reliability. The output of the light-curing unit was verified before each curing session using a radiometer (Demetron, Kerr Corporation, Orange, CA, USA) to confirm consistent intensity (450 ± 20 mW/cm²).

Statistical analysis

Data were analyzed using Statistical Package for Social Sciences (SPSS) version 26.0 (IBM Corp., Armonk, New York, USA). Normality was confirmed using the Shapiro-Wilk test (p > 0.05), confirming parametric assumptions. Continuous variables were reported as mean ± standard deviation (SD) with ranges and confidence intervals. Comparisons between the distance and time groups were performed using two-way analysis of variance (ANOVA), followed by Bonferroni post-hoc tests for pairwise comparisons. Statistical significance was set at p < 0.05.

## Results

Microhardness varied significantly with the curing distance and time. The results show a clear trend where the mean value increases with longer curing times for every distance group. At a 1 mm distance, the mean rises from 97.47 (40 s) to 105.30 (60 s) to 112.13 (80 s). Similarly, for 3 mm, it increases from 92.47 to 98.21 to 106.62. This pattern continues for 5 mm (89.22 to 91.07 to 98.66) and 8 mm (80.94 to 91.74 to 94.25). Furthermore, at any given curing time, the mean value is inversely related to the curing distance; the 1 mm group consistently yields the highest means, followed by 3 mm, 5 mm, and finally, 8 mm with the lowest values. The inference is that both a shorter curing distance and a longer curing time are critical factors for achieving a higher outcome. The most effective combination is the shortest distance (1 mm) with the longest time (80 s), while performance diminishes significantly with greater distance, even when using extended curing durations (Table 1).

**Table 1:** Descriptive statistics of microhardness (Vickers hardness number) for the stratified group and time Data are presented as mean ± standard deviation. Microhardness was measured using the VHN. Curing time is in seconds.

Groups based on curing distance	Curing time	95% confidence interval for mean (lower limit – upper limit)	Mean ± Standard Deviation
Group A (1 mm)	40 s	93.08 - 104.52	97.47 ± 4.60
	60 s	98.67 - 114.13	105.30 ± 6.23
	80 s	108.98 - 127.02	112.13 ± 4.85
Group B (3 mm)	40 s	89.44 - 96.16	92.47 ± 5.12
	60 s	86.16 - 102.64	98.21 ± 2.61
	80 s	89.46 - 118.14	106.62 ± 3.49
Group C (5 mm)	40 s	72.35 - 91.65	89.22 ± 3.74
	60 s	72.22 - 97.78	91.07 ± 2.24
	80 s	91.36 - 101.04	98.66 ± 3.90
Group D (8 mm)	40 s	72.51 - 87.89	80.94 ± 2.17
	60 s	85.34 - 95.46	91.74 ± 5.68
	80 s	91.29 - 103.11	94.25 ± 8.79

Two-way analysis of variance (ANOVA) revealed that the significant main effects of curing distance (p < 0.001) and curing time (p < 0.001) on the microhardness of the posterior composite, with curing distance contributing more substantially (96% variance explained). The interaction effect (distance × time) was not significant (p = 0.065), indicating that the impact of time on hardness was consistent across distances. The large effect sizes indicate that the curing distance (1-3 mm) and duration (≥40 s) are critical independent factors for optimal polymerization (Table 2).

**Table 2:** Results of two-way analysis of variance (ANOVA) for intergroup comparison of microhardness based on curing distance and curing time *p < 0.05 denotes statistical significance using two-way analysis of variance, df: degrees of freedom

Interaction factor	Type III sum of squares	df	Mean square	F value	p value	Effect size
Curing distance	24513.6	3	8171.2	353.86	< .001*	0.96
Curing timing	2794.13	2	1397.07	60.5	< .001*	0.72
Distance x Timing	297.6	6	49.6	2.15	0.065	0.21

The Bonferroni post-hoc analysis for microhardness across light-curing tip distances (1 mm, 3 mm, 5 mm, and 8 mm) and curing times (40 s, 60 s, and 80 s) revealed significant differences. The highest microhardness was obtained at 1 mm for 80 s, followed by 60 s and 40 s. Similar results were seen at 3 mm, where the highest microhardness was noted at 80 s. At 8 mm, the microhardness was the lowest. Longer exposure (80 s) consistently enhanced the microhardness, with a significant decline beyond 3 mm (Table 3).

**Table 3:** Statistically significant pairwise comparisons of microhardness values across different curing distances and curing times based on Bonferroni post-hoc analysis Only significant comparisons are shown; values include p-values and corresponding t-statistics. *p < 0.05 was considered statistically significant using Bonferroni post-hoc analysis.

Pairwise comparison	p value	t statistics
Group A (1 mm, 80 s) vs. Group B (3 mm, 40 s)	0.001*	17.64
Group A (1 mm, 80 s) vs. Group B (3 mm, 60 s)	0.001*	20.14
Group A (1 mm, 80 s) vs. Group C (5 mm, 40 s)	0.001*	24.94
Group A (1 mm, 80 s) vs. Group C (5 mm, 60 s)	0.001*	20.14
Group A (1 mm, 80 s) vs. Group D (8 mm, 40 s)	0.001*	24.94
Group A (1 mm, 80 s) vs. Group D (8 mm, 60 s)	0.001*	22.57
Group A (1 mm, 80 s) vs. Group D (8 mm, 80 s)	0.001*	19.35
Group B (3 mm, 80 s) vs. Group D (8 mm, 40 s)	0.001*	13.49
Group C (5 mm, 80 s) vs. Group D (8 mm, 40 s)	0.001*	13.49
Group C (5 mm, 80 s) vs. Group D (8 mm, 60 s)	0.01*	11.12
Group D (8 mm, 80 s) vs. Group B (3 mm, 40 s)	0.01*	7.90
Group D (8 mm, 80 s) vs. Group C (5 mm, 40 s)	0.01*	7.90

## Discussion

The findings of this in vitro study demonstrated that the curing tip distance and exposure time significantly influenced the microhardness of the SureFil™ posterior composite resin, with the curing distance exerting a more pronounced effect. The highest microhardness was observed in the 1 mm group at 80 s, whereas the lowest was recorded in the 8 mm group at 40 s. Longer curing times (80 s) consistently improved microhardness across all distances, with a notable increase at 5 mm. The non-significant interaction effect suggests that the impact of curing time on microhardness remained relatively consistent across distances.

Microhardness, a measure of the resistance of a material to surface deformation, is closely linked to the degree of conversion of the resin matrix, which determines the mechanical strength and durability of composite restorations. To establish the extent of cure through the analysis of hardness measurements at both the upper and lower surfaces, it is customary to compute the ratio of the lower to upper hardness.

Subsequently, an arbitrary threshold for this ratio is often determined to ascertain that the lower surface is sufficiently cured; values such as 0.80 and 0.85 have frequently been employed in this context [13,14]. Ferracane illustrated a strong relationship between an increase in hardness and an increased degree of conversion [15].

The inverse relationship between the curing tip distance and microhardness can be explained by the inverse square law, which states that light intensity decreases with the square of the distance from the source. At 1 mm, the light-curing unit delivered the maximum irradiance (450 mW/cm²), facilitating optimal monomer-to-polymer conversion and resulting in the highest VHN. As the distance increased to 8 mm, the light intensity significantly reduced, leading to incomplete polymerization and lower microhardness. This finding is consistent with the findings of Oh et al. and Marovic et al., who reported that an increased curing distance reduces light intensity, lowers the degree of conversion, and compromises mechanical properties such as microhardness [16,17].

The significant effect of exposure time on microhardness underscores the importance of sufficient energy delivery for polymerization. At 80 s, all groups exhibited higher microhardness than at 40 s, particularly at greater distances. This can be attributed to the cumulative energy (radiant exposure, measured in J/cm²) delivered to the composite, which is the product of irradiance and time. Previous studies have documented that an increase in exposure time leads to an increase in microhardness, particularly on the top surface [18,19]. Longer exposure times allowed more photoinitiators, such as camphorquinone in SureFil™, to be activated, promoting greater cross-linking within the resin matrix. Barakah recommended a longer exposure time with increasing curing distance [4]. This explains the improved microhardness observed at 80 s across all distances. However, the diminishing returns at 8 mm suggest a threshold beyond which increased exposure time cannot fully compensate for reduced light intensity owing to distance [16].

At 1 and 3 mm, significant differences in microhardness were observed across all curing times, indicating that proximity enhances the efficacy of longer curing. At 5 mm, the lack of significant differences suggests that light attenuation begins to limit polymerization efficiency, even with extended exposure. At 8 mm, the consistently low microhardness across all curing times highlights the severe impact of reduced irradiance, corroborating the findings of Oh et al., who reported that curing distances beyond 8 mm significantly impair the composite properties because of insufficient light penetration [16]. According to Okuse et al., the depth of cure was significantly greater when the light-curing tip was positioned at 0 mm and least at 8 mm [20].

The non-significant interaction effect indicates that the curing time and distance act as independent factors, with distance having a greater influence. This is likely because the light intensity, governed by distance, is the primary determinant of the number of photons available to initiate polymerization [21,22]. At greater distances, even extended curing times could not fully overcome the reduced photon flux, as evidenced by the low VHN at 8 mm. This aligns with a previous review, which emphasized that irradiance is a critical factor in achieving adequate polymerization, with exposure time playing a secondary role when the intensity is suboptimal [23].

The choice of SureFil™, a packable posterior composite with 82% filler loading and a 0.8-micron particle size, may also influence these results. A high filler content increases light scattering within the material, potentially reducing light penetration into deeper layers, particularly at greater curing distances [23]. Leprince et al. noted that composites with high filler loads require higher radiant exposure to achieve optimal polymerization, and polymerization depends on the filler content, which explains why longer curing times (80 s) were more effective, particularly at 1-3 mm distances [24]. The study’s use of a halogen light-curing unit (450 mW/cm²) likely resulted in greater light attenuation compared to modern high-intensity light-emitting diode units, though extended curing times still supported improved microhardness in the tested samples.

These findings have significant implications for clinical practice. Based on the findings of this study using a halogen curing unit and SureFil™ composite, optimal microhardness was achieved with a minimal tip distance (1-3 mm) and extended curing times (60-80 seconds). These specific parameters are recommended for similar conditions, but may differ with LED lights, other resin types, or higher irradiance units. These parameters ensure adequate polymerization, reducing the risk of wear, marginal breakdown, or secondary caries. Clinicians should also verify the output of their light-curing units regularly, using a radiometer to ensure consistent irradiance, as variations can compromise the outcomes. For Class II restorations, where access may be challenging, the use of sectional matrix systems or transparent matrices can help maintain proximity and optimize light delivery.

This study has several limitations. As an in vitro investigation, it does not account for clinical variables, such as saliva contamination, operator variability, or patient movement, which may affect curing efficacy. The use of a single composite and light-curing unit limits generalizability, as different materials and curing units may respond differently to the curing distance and time. The focus of this study on surface microhardness may not fully reflect the mechanical properties of deeper layers, where light penetration is further attenuated. Additionally, a maximum curing distance of 8 mm may not represent extreme clinical scenarios, and longer distances could further elucidate the limits of polymerization. Finally, the study did not assess other properties, such as wear resistance or bond strength, which are critical for restoration longevity. Future research should explore these factors in various clinical settings and using different composite formulations.

## Conclusions

This in vitro study demonstrated that the curing tip distance and exposure time significantly influence the microhardness of the posterior composite resin. Optimal microhardness was achieved with the closest curing distance and longest exposure time, whereas microhardness decreased notably with greater distances and shorter curing times. Further analysis confirmed the significant main effects of both curing distance and exposure time, with curing distance being the dominant factor, and a non-significant interaction effect, indicating the independent contributions of these factors to polymerization. These findings emphasize the importance of minimizing the curing tip distance and ensuring a sufficient exposure time in clinical practice to optimize the mechanical properties and durability of posterior composite restorations.
